# Enhancing organizational readiness for implementation: constructing a typology of readiness-development strategies using a modified Delphi process

**DOI:** 10.1186/s13012-021-01132-0

**Published:** 2021-06-10

**Authors:** Sigal Vax, Marianne Farkas, Zlatka Russinova, Kim T. Mueser, Mari-Lynn Drainoni

**Affiliations:** 1 Rehabilitation Sciences Program, College of Health & Rehabilitation, Sargent College, Boston University, 940 Commonwealth Ave W, Boston, MA 02215 USA; 2 Center for Psychiatric Rehabilitation, College of Health & Rehabilitation, Sargent College, Boston University, Boston, MA USA; 3 Section of Infectious Diseases, Department of Medicine, Boston University School of Medicine, Boston, MA USA; 4 Department of Health Law Policy & Management, Boston University School of Public Health, Boston, MA USA

**Keywords:** Implementation strategies, Pre-implementation, Transtheoretical model, Stage-based, Evidence-based practices, Organizational readiness for change, Modified Delphi

## Abstract

**Background:**

Knowledge about the development of organizational readiness for implementation (ORI) is limited. ORI, referred to as the willingness and capacity of all relevant stakeholders to change practice, is critical for increasing the adoption rate of evidence-based practices and improving implementation outcomes. However, no methodology currently guides ORI’s enhancement or addresses differences in readiness needs across an organization. This study used the transtheoretical model (TTM) as a framework for classifying a well-established compilation of implementation strategies into three readiness stages: pre-contemplation, contemplation, and preparation.

**Methods:**

A modified Delphi method was used to establish consensus among a panel of purposefully selected research and field implementation experts. The Delphi process involved three rounds of online questionnaires. The third round also included a live video discussion to clarify definitions in an effort to increase consensus among experts.

**Results:**

Of the 73 strategies reviewed, the experts identified 75% (*n* = 55) as relevant for pre-implementation and reached a high-level agreement on the assignment of 7% (*n* = 5) of the strategies to the pre-contemplation stage (ORI-1), 25% (*n* = 18) to the contemplation stage (ORI-2), and 52% (*n* = 38) to the preparation stage (ORI-3). Several strategies were identified as relevant to more than one stage.

**Conclusions:**

Participating experts were able to reach high-level agreement on the relevance of specific sets of implementation strategies to each of the three ORI stages. The lowest number of strategies was assigned to ORI-1 and the highest number to ORI-3. Given the overlap of strategies across ORI stages, there is a need to better understand the specific utilization of such strategies at different stages. Future studies are needed to empirically evaluate the relevance and applicability of this expert-informed typology based on implementers’ experiences in the field.

**Supplementary Information:**

The online version contains supplementary material available at 10.1186/s13012-021-01132-0.

Contributions to the literature

- Organizational readiness for implementation improves the adoption rate of health and rehabilitation practices. However, knowledge is limited as to how organizational readiness may be enhanced prior to implementation.
- In this study, a panel of research and field implementation experts identified strategies relevant for readiness development and organized them into three stages occurring pre-implementation: pre-contemplation, contemplation, and preparation.
- This study informed the development of a systematic framework for addressing different readiness needs across an organization. The results improve our understanding of how pre-implementation strategies may be used to increase the adoption of evidence-based practices and improve health and rehabilitation outcomes.

## Background

Organizational readiness for implementation (ORI) is a complex construct encompassing both the willingness and perceived capacity of stakeholders across an organization to engage in adopting a new practice [1–5]. ORI significantly impacts the adoption rates of evidence-based practices [6–12], which lead to improved health and rehabilitation services [13]. Organizational-level factors influencing ORI include organizational climate and resources [14], while individual-level factors constitute attitudes, commitment, and self-efficacy to execute the change [1, 2]. Multiple assessment tools have been developed to measure different aspects of ORI at the individual and collective levels across an organization [15–19]. However, it is unclear how readiness may be enhanced when found insufficient to ensure successful implementation.

Given this gap, there is a need to determine which implementation strategies could be most helpful prior to implementation and to plan for their systematic utilization. The Expert Recommendations for Implementing Change (ERIC) project collated a list of 73 strategies defined by leading experts in implementation science [20]. Follow-up studies have organized this list of strategies into sub-categories related to their primary function (e.g., support clinicians) [21] or the contextual barriers they address (e.g., available resources) [22]. However, these follow-up studies have not specified the optimal timing for using each strategy, nor do they identify which strategies are relevant for readiness development.

The functional classification of implementation strategies was first combined with a temporal measure by Bunger et al. [23], who found that many strategies were used throughout implementation as ongoing activities, but their utilization differed between pre-implementation and active implementation phases. Specifying the pre-implementation utilization of discrete strategies and their expected readiness outcomes could inform a methodology for ORI development and add to these strategies’ usability [24].

The transtheoretical model (TTM) [25] is a framework developed to support individuals in changing persistent behaviors, such as drug and alcohol abuse [26–28], smoking [29, 30], and gambling [31]. The TTM was adapted to facilitate organizational change [32], including changes in policies and practices in various organizations [33–40]. This model focuses on changing interest, attitudes, and beliefs of individuals within the organization regarding an expected change, therefore aligns with current definitions of organizational readiness for change [1, 2]. The TTM is comprised of five stages of behavioral change: (1) pre-contemplation, (2) contemplation, (3) preparation, (4) action, and (5) sustainment. Individuals within an organization may proceed through these stages at a different pace as they embrace and sustain a behavioral change related to their work practice. Recent studies have demonstrated the usability of the TTM as a framework for organizing implementation strategies [5, 39]. Using this framework to organize the comprehensive and widely used list of ERIC strategies offers a systematized approach to ORI development and adds specificity to the ERIC strategies.

The purpose of the current study was to construct a stage-based typology of ORI development strategies taken from the ERIC Project. Our goal was to reach a consensus among a group of implementation experts on strategies relevant for pre-implementation and their stage classification into the TTM readiness stages—pre-contemplation, contemplation, and preparation. A modified Delphi process [41] was used to build the experts’ consensus and reach an initial ORI typology.

## Methods

### Delphi participants

A panel of implementation experts was recruited to participate in a three-round modified Delphi process. The experts represented two groups: (1) research experts, selected from a review of the Implementation Science Journal between the years 2009–2020, and (2) field experts, selected from a pool of implementation leaders from the community mental health (CMH) field known to the authors. The inclusion criterion for research experts was that they had published two or more peer-reviewed articles in the last 10 years specifying a model or a framework related to organizational readiness, stages of implementation, or strategies for implementation. Field experts included administrators and implementation consultants with at least 10 years of experience in leading implementation efforts of one or more evidence-based practices in the CMH field. The participants were recruited only from the USA to allow better coordination during the consensus-building process.

Nineteen research experts and 11 field experts were identified. Eight of them were excluded as they collaborated on research or field projects with other, more senior participants on the list. Thus, an email describing the study, expected tasks, and timeline was sent to 22 identified experts—17 researchers and five field experts. Eleven of the research experts and all five field experts agreed to participate in the study. The research experts represented different health-related fields (e.g., public health, health policy, behavioral health, children’s mental health) and included two panelists who developed a theory, or a framework related to organizational readiness, four who developed models related to stages of implementation, and five that focused their research on strategies for implementation. A full list of the panelists and their affiliations can be found in the “Contributors” section.

### Modified Delphi process

The Delphi method is widely used in health research to develop consensus on group opinion [41]. Delphi methods are usually used to address complex, large, multidisciplinary problems where knowledge is incomplete or when uncertainty and lack of evidence exist [42, 43]. The modified Delphi method aims to reach agreement among a group of experts on a set of selected items [41, 44], rather than elicit agreement about an open question, which is the focus of the original Delphi process [45]. We chose to use the modified Delphi process since the ERIC compilation of strategies provides a pre-defined set of items. In addition, this version of the Delphi method typically improves the response rate and provides a solid grounding for previously developed work [44]. The modified Delphi method has been used in several projects to define constructs and processes related to implementation [15, 20, 46–48]. It is considered a highly efficient method to promote conceptualization in the implementation science field [49].

**Fig. 1:**
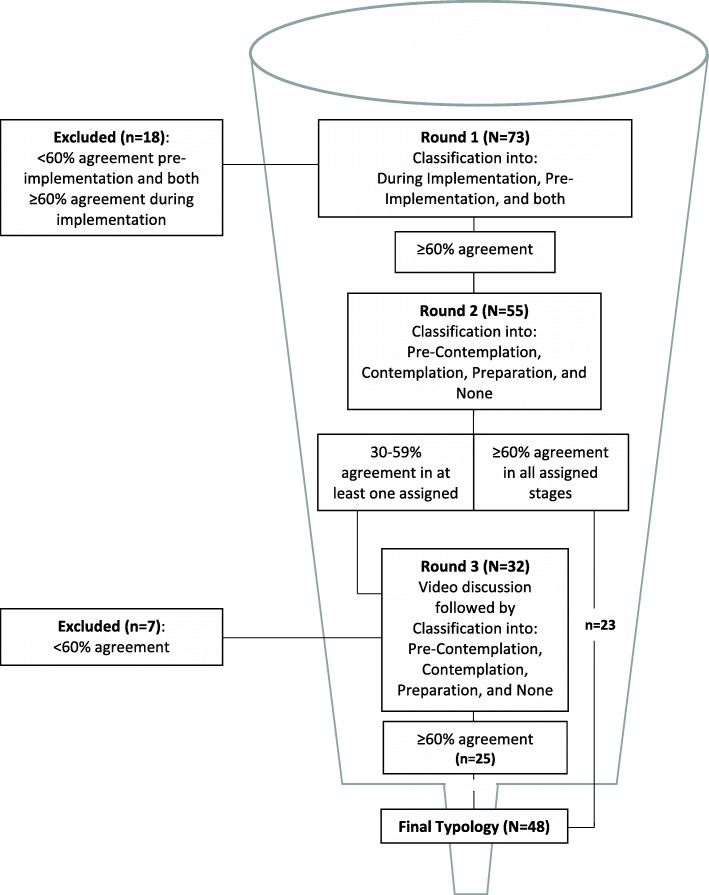
Flowchart of the Delphi process

In this study, the modified Delphi process included three rounds to develop consensus among the experts on the categorization of ERIC implementation strategies into ORI stages. The experts were not given the opportunity to add or modify strategies to avoid another consensus process related to these changes. The first two rounds were conducted individually, using an online questionnaire; the third round involved a video conference of all the panelists, followed by another individual questionnaire. The panelists had 2 weeks to complete each round with a week break between the submission deadline and the launch of the next round. The rapid turnaround was designed to maintain participants’ engagement and reduce attrition [50]. The overall process took less than 3 months, with the first round released on June 1, 2020, and the third round completed on August 20, 2020. Consistent with the literature [51], we set a 60% agreement threshold, which represented the majority of votes, to determine high-level agreement among the participants at each round. Medium- and low-level agreements were set to 30–59% and under 30%, respectively. The consensus-building process was designed like a funnel, offering more granularity from one step to another (see Fig. 1). The high-level agreement served as the inclusion criterion into the typology. The medium-level agreement was only used in round 2 to identify strategies agreed by the experts as relevant during pre-implementation but lacking consensus regarding their stage-classification. .

The study was exempted by the Boston University Charles River Campus Institutional Review Board. Nevertheless, participant confidentiality was maintained in the survey rounds to avoid any pressure between co-panelists, as well as to guarantee the same weight for all participants’ responses [45]. The participants were only exposed to each other in the final round during the video conference discussion.

#### Round 1

A link to an online questionnaire was emailed to all the participants via Qualtrics, a secure survey platform. The panelists were asked to identify strategies they perceived as relevant for pre-implementation from the full list of the ERIC strategies (N=73); definitions for these strategies were included [20]. Participants were asked to check off one category in which they perceived the strategy to be most relevant: (1) pre-implementation, (2) during implementation, or (3) pre- and during implementation. Participants were provided with the definition for each stage: Pre-implementation was defined as “when members of the organization are considering changing their practice or preparing to engage in the implementation activities (e.g., evaluating the need to change, weighing the cost and benefits, and planning).” During implementation was defined as “when members of the organization are actively engaged in changing their practice or acquiring a new practice (e.g., training, supervision, problem-solving, and dissemination activities).” At the end of this round, strategies endorsed by the majority of the participants (≥60%) as relevant for pre-implementation only, or both pre- and during implementation, were compiled together and carried forward to the second round.

#### Round 2

The online questionnaire was revised to include only strategies that were carried over from the previous round. Participants were asked to indicate which stages were relevant for each strategy, or no stage at all (none category). The stages were defined based on the TTM definitions [52] as follows: ORI-1. Pre-contemplation—members of the organization have no awareness of the new practice or feel no pressing need to change their existing practice. Strategies to help members consider the new practice relate to generating inspiration for the change, anxiety about maintaining the status quo, communicating information about the change and how it can improve the organizational success and climate, or displaying strong leadership commitment to the change; ORI-2. Contemplation—the benefits of changing the practice are recognized but are still outweighed by the potential risks or costs. Strategies to be used in this stage mostly relate to clarifying or modifying personal values and goals with respect to the change initiative; ORI-3. Preparation—interest and motivation for changing the practice have been established, and people are ready to create a plan or take small steps toward launching implementation activities. Strategies in this stage mostly relate to planning the implementation process, encouraging involvement, and empowering members of the organization to take key positions in the implementation process.

Strategies that reached a high-level agreement (≥60%) for all ORI stages to which they were assigned were included in the final typology. Strategies that reached a medium-level agreement (30–59%) in at least one category were carried forward to the third round to develop better consensus about their assignment.

#### Round 3

The final round consisted of two steps aimed at building consensus around the assignment of strategies with medium-level agreement and finalizing the ORI typology. First, a 90-min live video conference was conducted using the Zoom platform to clarify and refine the decision process related to strategy classification. The participants were presented examples of strategies representing four different types of disagreement: (1) high-level agreement on one stage and medium-level agreement on other stages; (2) medium-level agreement on all ORI stages; (3) a mix of low- and medium-level agreement; and (4) medium-level agreement on the none category. Using these examples, the experts engaged in a structured discussion in which they reviewed the ORI stages, agreed on specific issues related to the decision process, and received guidance before the final classification task.

Second, a third and final questionnaire was sent via email containing all the medium-agreement level strategies that were carried over from round 2. Experts were asked to assign the strategies again to each ORI stage independent of their responses in the previous round. Strategies from round 2 with high-level agreement in all assigned stages were then combined with strategies that reached a high-level agreement in round 3 to create the final typology.

## Results

Sixteen panelists completed round 1, 15 completed round 2, and 13 completed round 3. The balance between research and field experts was maintained as two research experts and only one field expert dropped out.

### Round 1

The panelists sorted the 73 implementation strategies listed in the ERIC project into three categories of pre-implementation, during implementation, or both. Of the 73 strategies, the experts identified 55 (75%) as relevant to pre- and during implementation. Of the remaining 18 strategies, six (33%) were deemed relevant only during implementation, and twelve (67%) did not reach the agreement threshold for pre- and during implementation. These 18 strategies were excluded from further discussion. The 55 strategies considered relevant for pre-implementation were carried over to the next round.

### Round 2

Of the 55 strategies relevant for pre-implementation included in this round, five (9%) reached a high-level agreement as relevant to pre-contemplation (ORI-1), 19 (35%) to contemplation (ORI-2), and 47 (85%) to preparation (ORI-3). All the strategies assigned to pre-contemplation or contemplation with a high-level agreement were assigned to at least one other stage. Twenty-three (42%) strategies reached a high-level agreement for all stages for which they were allocated. Three of them were assigned to pre-contemplation, 10 to contemplation, and 21 to preparation. For example, “Develop a formal implementation blueprint” had a high-level agreement for preparation, and “Conduct needs assessment” had a high-level agreement for both pre-contemplation and contemplation. All 23 strategies that reached a high-level agreement were considered for inclusion in the ORI typology.

Twenty-eight strategies (51%) reached a high-level agreement for one stage and a medium-level agreement for another. For example, “Use data experts” had a high-level agreement as relevant for preparation, but a medium-level agreement for contemplation. Four more strategies (7%) reached only a medium-level agreement for one or more category. None of the 55 strategies included in this round had only low-level agreement. Thus, all 32 strategies with inconsistent or medium-level agreement were carried over to round 3 to determine their final allocation.

### Round 3

Participants in the live discussion generated three main guidelines regarding the conceptualization of intervention strategies used in different ORI stages. (1) The strategies should be viewed in the context of implementing relatively new practices, which present more readiness challenges, and not in the context of scaling up already disseminated interventions, for which there is usually more acceptance. (2) Some pre-implementation strategies are related to the external context (e.g., policy) or the intervention design (e.g., the “packaging” of the intervention); therefore, they are not relevant for organizational readiness development and should be assigned to the “None” category. (3) Some strategies can be used in different ways (like funding/contracting) at different stages, depending on the system in which the organization operates. Such strategies can be assigned to more than one stage, given the aim of developing a typology of strategies for people to choose from, based on their local needs or system structure.

At the end of the third round, 25 of the 32 strategies (78%) reached a high-level agreement in one or more stages (2 for pre-contemplation, 8 for contemplation, 17 for preparation) and were included in the final typology. Of the 25 strategies that reached a high-level agreement in this round, 14 (44%) retained their high-level allocation from the previous round and 11 (34%) had a shift in their allocation. Of those 11 strategies, four (36%) moved their high-level agreement from one stage to another (e.g., “Assess for readiness and identify barriers and facilitators” moved from preparation to contemplation). Six strategies (55%) shifted from high-level agreement in two stages to only one stage (e.g., “Conduct educational outreach visits” was previously assigned to both ORI-1 and 2, and after round 3 was only assigned to contemplation). One strategy (9%) had a high-level agreement added in another stage after having only one in the previous round (“Use an implementation advisor” was previously assigned to preparation and in this round was added to contemplation as well). The four strategies with only medium-level agreement in the previous round kept their status, and three more strategies were either removed from pre-implementation or their level of agreement decreased. Those seven strategies (21.5%) were eliminated from the typology.

### Final typology

Of the 73 implementation strategies identified by the ERIC project, the final expert-informed typology included 48 (66%) strategies deemed relevant to enhancing ORI prior to implementation. This typology was comprised of the 23 strategies from round 2 and the 25 strategies from round 3 that reached high-level agreement. The distribution of the 48 strategies was as follows: five strategies in pre-contemplation (10%), 18 in contemplation (37%), and 38 in preparation (79%). Some strategies were assigned to more than one stage. Four out of five strategies (80%) in pre-contemplation were also relevant to contemplation (e.g., “Conduct local consensus discussions”). Nine out of 18 strategies (50%) in contemplation were also relevant to preparation (e.g., “Build a coalition”). Only one strategy reached a high-level agreement in all three stages: “Conduct educational meetings.” Table 1 presents the strategies that reached a high-level agreement at the end of the Delphi process related to their stage-assignment. A more detailed presentation of the results for each classification category can be found in the Supplement 2 document.

**Table 1:** Final ORI typology

ORI-1 Pre-contemplation	ORI-2 Contemplation	ORI-3 Preparation	
**Unique to this stage** 1. Develop educational materials **Overlap with ORI-2** 2. Conduct local consensus discussions 3. Conduct local needs assessment 4. Inform local opinion leaders **Overlap across all stages** 5. Conduct educational meetings	**Unique to this stage** 1. Assess for readiness and identify barriers and facilitators 2. Conduct educational outreach visits 3. Fund and contract for clinical innovation 4. Identify and prepare champions 5. Identify early adopters 6. Visit other sites **Overlap with ORI-1** 7. Conduct local consensus discussions 8. Conduct local needs assessment 9. Inform local opinion leaders **Overlap with ORI-3** 10. Access new funding 11. Alter incentive/allowance structures 12. Build a coalition 13. Promote adaptability 14. Recruit, designate, and train for leadership 15. Shadow other experts 16. Use advisory boards and workgroups 17. Use an implementation adviser **Overlap across all stages** 18. Conduct educational meetings	**Unique to this stage** 1. Centralize technical assistance 2. Change accreditation or membership requirements 3. Change physical structure and equipment 4. Change record system 5. Create new clinical teams 6. Create or change credentialing and/or licensure standards 7. Develop a formal implementation blueprint 8. Develop an implementation glossary 9. Develop and implement tools for quality monitoring 10. Develop and organize quality monitoring systems 11. Develop disincentives 12. Develop resource sharing agreements 13. Distribute educational materials 14. Involve patients/consumers and family members 15. Make billing easier 16. Make training dynamic 17. Model and simulate change 18. Obtain formal commitments 19. Place innovation on fee for service lists/formularies	20. Prepare patients/consumers to be active participants 21. Promote network weaving 22. Revise professional roles 23. Stage implementation scale up 24. Tailor strategies 25. Use data experts 26. Use data warehousing techniques 27. Use train the trainer strategies 28. Work with educational institutions **Overlap with ORI-2** 29. Access new funding 30. Alter incentive/allowance structures 31. Alter patient/consumer fees 32. Build a coalition 33. Promote adaptability 34. Recruit, designate, and train for leadership 35. Shadow other experts 36. Use advisory boards and workgroups 37. Use an implementation adviser **Overlap across all stages** 38. Conduct educational meetings

## Discussion

This study aimed to facilitate the adoption of new practices by developing a typology of pre-implementation strategies to address ORI enhancement. We used the TTM as the theoretical framework to break down the construct of organizational readiness into a stage-based process. Using a modified Delphi method, we built consensus among implementation experts regarding the classification of the ERIC strategies into three readiness stages: pre-contemplation (ORI-1), contemplation (ORI-2), and preparation (ORI-3). The experts identified 48 strategies as relevant for pre-implementation readiness development and specified which strategies were most appropriate for each ORI stage.

The study confirms that implementation strategies can be linked to specific readiness stages, as defined by the TTM, and aligns with the recommendation to develop guidelines for tailoring implementation strategies from the ERIC compilation [20]. Our typology may be used in conjunction with the ERIC compilation to inform the selection and utilization of specific strategies to address readiness needs in a directed and practical way. Other attempts to categorize and specify the utilization of the ERIC strategies have lacked a temporal dimension [21, 22, 53]. We have addressed this gap by providing a process-based approach that distinguishes between pre-implementation and during implementation phases as well as provide steps to be taken prior to implementation. Future studies may replicate our work to specify strategies from the ERIC compilation relevant during active implementation and sustainment to complete the association between all five stages of the TTM and the ERIC list.

Furthermore, the TTM provides a framework to address the critical psychological aspects of attitudes and beliefs towards the change. While those implementing a new approach may include “early adopters” or those eager to implement an innovation [54], a larger proportion of people fall into the pre-contemplation and contemplation stages when introduced to a new change process [32, 55]. Although several researchers have emphasized the importance of individuals’ positive attitudes and beliefs as antecedents to organizational readiness [4, 56–58], specific strategies for enhancing these attitudes and beliefs have been previously unaddressed [4, 17, 59]. The conceptual structure of pre-contemplation, contemplation, and preparation provides a refined view of the different psychological barriers and readiness needs that can more accurately direct enhancement efforts. Linking readiness needs with discrete strategies can help respond to deficits identified in readiness assessments. For example, the TCU-ORC [60], the most established readiness measurement tool [17], includes measures related to individuals’ awareness of pressures for change. If this assessment reveals lack of knowledge and understanding of the need to change, pre-contemplation strategies can be used to address it. Focusing on the psychological barriers of individuals in organizations at the early stages of pre-contemplation and contemplation can increase the number of engaged participants in the preparation and action stages, leading to a greater positive impact on the change process.

While our typology contains specific sets of implementation strategies for each ORI stage, the number of strategies assigned to ORI-3 was much larger than the number of strategies assigned to ORI-1 and ORI-2. Two explanations might help clarify this difference between stages. First, it may be easier to operationalize and measure preparation-related strategies than contemplation and pre-contemplation strategies due to their more practical nature. For example, “Distribute educational materials” describes a concrete task, while “Identify early adopters” is more generic and calls for more specification as to how it should be done.

Another explanation lies in how the strategies are described. It has been recognized that the implementation strategies listed in the ERIC compilation vary in their level of specificity [20, 22, 23, 53]. This variability makes it difficult to compare the number of strategies in one stage with another, as it is possible to have fewer but more broadly defined strategies in one stage, and a greater number of more specific strategies in another stage. A similar concern was raised by several panelists in our study, regarding their ability to uniquely classify some of the strategies involving multiple activities that span across stages. Further investigation showed that these broad strategies were mostly assigned to ORI-1 and ORI-2, while the strategies assigned to ORI-3 were more concrete. To resolve the unbalanced distribution of strategies and create a similar degree of specificity across strategies, we suggest that future work focus on breaking down some of the broad strategies in ORI-1 and 2. For example, “Conduct needs assessment,” appearing in ORI-1 and ORI-2, can be divided into “Assess client needs,” which is more relevant for establishing the need to change practice during ORI-1, and “Assess staff needs for support,” which may be more appropriate in ORI-2. To date, studies related to implementation strategies have mostly focused on clustering “small” strategies [20, 21]. More information is needed on how “broad” strategies can be dismantled to make them more specific. Exploring the utility of this typology in the real world will provide valuable information about how strategies can be divided or collapsed.

The overlap of strategies between stages may also be attributed to the broad nature of some of the strategies. However, another possible explanation for the overlap is the experts’ decision to assign strategies to more than one stage, if needed. Several experts argued that some of the strategies could be used differently, depending on the context in which the change process occurs. For example, “Inform local opinion leaders” was assigned to both ORI-1 and 2 with the notion that it can be utilized in both stages, depending on local opinion leaders’ role, impact, and relationship with the organization. The panel decided that since the typology could be utilized in a wide range of organizational contexts, it would be better to assign strategies to multiple stages and allow implementers to “pick and choose” based on their fit with the local setting. Future studies could help clarify the circumstances in which same strategies are used for different stages.

Our confidence in the results is limited to the experts who agreed to participate in the study. The experts we identified as relevant to our topic that could not participate might have provided other perspectives or strengthened our results even further. Avella [45] states that typical Delphi panels range between 10 and 100 members and usually consist of two or three expert groups, depending on the relevant stakeholders to provide input on the research question. While our panel represented diverse perspectives from both academia and the field, additional implementers representing frontline roles could have provided valuable user experience [61]. Additionally, our panel included only US-based experts and did not account for geographical or cultural representation. This approach may have downplayed the impact of cultural differences on the structure of the typology. While the choice of limiting our panel to US experts helped us overcome the technical challenges of time differences and language obstacles, the lack of international input may limit the generalizability of the typology. Future studies should strive to create panels reflecting more diverse geographic and cultural contexts for implementation, using focus groups rather than large-scale discussion meetings to accommodate different time zones, cultural differences, and professional backgrounds.

Another limitation of the study is that our list of implementation strategies included only those identified in the ERIC project and did not include additional strategies that might be missing in the literature. The ERIC compilation of strategies is the most comprehensive list to date. It includes a definition for each strategy that was established in a consensus-building process involving a large panel of implementation experts. We deliberately avoided asking the experts in our study to offer additional strategies beyond those listed in the ERIC compilation to refrain from needing another process of agreement on strategies and their definitions that might not be consistent with the ERIC list. One potential next step would be to conduct a field study to elicit additional implementation strategies and how they were used relative to the TTM stages. Additional work can be done to identify other refinements and sub-constructs already suggested for the ERIC compilation (e.g., [21, 22, 53]) to include them in the typology and provide a more holistic view of current knowledge.

The results from this study provide an initial framework to systematically address ORI enhancement, with the goal of positively impacting the adoption rate of evidence-based practices. Further refinement and development of pre-implementation strategies, specifically those addressing pre-contemplation and contemplation, could help increase ORI levels in organizations that might otherwise avoid joining an implementation project.

## Conclusion

This study was an initial effort to specify the possible utilization of implementation strategies along stages of readiness development. The typology constructed in the study presents a framework with greater specificity concerning how the ERIC project strategies can be used to support the implementation outcome of organizational readiness for implementation. However, while the participating experts were able to agree on distinct sets of strategies suitable for each ORI stage, further specificity is needed, especially for strategies that affect attitudes and beliefs in the pre-contemplation and contemplation stages. It is also important to test this typology in the field, both as an exploration of its utility and as a way of understanding more about the different uses of similar strategies across ORI stages.

## Contributors

We would like to acknowledge each member of the expert panel, listed here by alphabetical order of their last name: Gregory Aarons, University of California, San Diego; Cayte Anderson, University of Wisconsin-Madison; Deborah Becker, The IPS Employment Center at the Rockville Institute, Westat; Torrey A. Creed, University of Pennsylvania; Lisa Dixon, Columbia University; Rani Elwy, Brown University; Joseph Marrone, Boston University and UMASS Boston; Janice Prochaska, Prochaska Change Consultants; Lisa Razzano, University of Chicago, and Thresholds Inc., Illinois; Lisa Saldana, Oregon Social Learning Center (OSLC); Virginia Selleck, Private consultant; Christopher Shea, University of North Carolina-Chapel Hill; Thomas Waltz, Eastern Michigan University; Abe Wandersman, The Wandersman Center and the University of South Carolina-Columbia; Bryan Weiner, University of Washington; Nathaniel Williams, Boise State University.

## Supplementary Information

**Additional file 1.** ORI Delphi_Supplement 1_CREDES Reporting Checklist.PDF – CREDES reporting checklist for a Delphi study.

**Additional file 2.** ORI Delphi_Supplement 2_Results.PDF – A summary of the results from Round 2 and Round 3.

## Data Availability

The datasets used and/or analyzed during the current study are available from the corresponding author on reasonable request.

## Abbreviations

ORI: Organizational readiness for implementation
TTM: Transtheoretical Model
ERIC: Expert Recommendations for Implementing Change
CMH: Community mental health

## References

[1] Weiner BJ (2009). A theory of organizational readiness for change. Implement Sci.

[2] Holt DT, Vardaman JM (2013). Toward a comprehensive understanding of readiness for change: the case for an expanded conceptualization. J Chang Manag.

[3] Benzer JK, Charns MP, Hamdan S, Afable M (2017). The role of organizational structure in readiness for change: a conceptual integration. Health Serv Manag Res.

[4] Rafferty AE, Jimmieson NL, Armenakis AA (2013). Change readiness: a multilevel review. J Manag.

[5] Vax S, Gidugu V, Farkas M, Drainoni M-L (2021). Ready to roll: strategies and actions to enhance organizational readiness for implementation in community mental health. Implement Res Pract.

[6] Powell BJ, Proctor EK, Glass JE (2014). A systematic review of strategies for implementing empirically supported mental health interventions. Res Soc Work Pract.

[7] Simpson DD, Flynn PM (2007). Moving innovations into treatment: a stage-based approach to program change. J Subst Abus Treat.

[8] Weiner BJ, Amich H, Lee S-YD (2008). Conceptualization and measurement of organizational readiness for change: a review of the literature in health services research and other fields. Med Care Res Rev.

[9] Malthe Bach-Mortensen A, Lange BCL, Montgomery P (2018). Barriers and facilitators to implementing evidence-based interventions among third sector organisations: a systematic review. Implement Sci.

[10] Hagedorn HJ, Heideman PW (2010). The relationship between baseline organizational readiness to change assessment subscale scores and implementation of hepatitis prevention services in substance use disorders treatment clinics: a case study. Implement Sci.

[11] Lundgren L, Amodeo M, Chassler D, Krull I, Sullivan L (2013). Organizational readiness for change in community-based addiction treatment programs and adherence in implementing evidence-based practices: a national study. J Subst Abus Treat.

[12] Williams NJ (2016). Multilevel mechanisms of implementation strategies in mental health: integrating theory, research, and practice. Adm Policy Ment Health Ment Health Serv Res.

[13] Proctor EK, Silmere H, Raghavan R, Hovmand P, Aarons G, Bunger A, Griffey R, Hensley M (2011). Outcomes for implementation research: conceptual distinctions, measurement challenges, and research agenda. Adm Policy Ment Health Ment Heal Serv Res.

[14] Castañeda SF, Holscher J, Mumman MK, Salgado H, Keir KB, Foster-Fishman PG (2012). Dimensions of community and organizational readiness for change. Prog Community Health Partnerships Res Educ Action.

[15] Timmings C, Khan S, Moore JE, Marquez C, Pyka K, Straus SE (2016). Ready, Set, Change! Development and usability testing of an online readiness for change decision support tool for healthcare organizations. BMC Med Inform Decis Mak.

[16] Allen JD, Towne SD, Maxwell AE, Dimartino L, Leyva B, Bowen DJ (2017). Measures of organizational characteristics associated with adoption and/or implementation of innovations: a systematic review. BMC Health Serv Res.

[17] Weiner BJ, Mettert KD, Dorsey CN, Nolen EA, Stanick C, Powell BJ, Lewis CC (2020). Measuring readiness for implementation: a systematic review of measures’ psychometric and pragmatic properties. Implement Res Pract.

[18] Sanders KA, Wolcott MD, McLaughlin JE, Shea CM, Pinelli NR. Organizational readiness for change: preceptor perceptions regarding early immersion of student pharmacists in health-system practice. Res Soc Adm Pharm. 2017; [cited 2017 Apr 29]. Available from: http://ac.els-cdn.com.ezproxy.bu.edu/S1551741116303230/1-s2.0-S1551741116303230-main.pdf?_tid=6de41fa8-2cf8-11e7-bde3-00000aacb35f&acdnat=1493483296_b8c221441cc2b14ed33e4430b6d84567. 10.1016/j.sapharm.2017.03.004 28356213

[19] Aarons GA, Glisson C, Hoagwood K, Kelleher KK, Landsverk J, Cafri G (2010). Psychometric properties and U.S. national norms of the Evidence-Based Practice Attitude Scale (EBPAS). Psychol Assess.

[20] Powell BJ, Waltz TJ, Chinman MJ, Damschroder LJ, Smith JL, Matthieu MM (2015). A refined compilation of implementation strategies: results from the Expert Recommendations for Implementing Change (ERIC) project. Implement Sci.

[21] Waltz TJ, Powell BJ, Matthieu MM, Damschroder LJ, Chinman MJ, Smith JL (2015). Use of concept mapping to characterize relationships among implementation strategies and assess their feasibility and importance: results from the Expert Recommendations for Implementing Change (ERIC) study. Implement Sci.

[22] Waltz TJ, Powell BJ, Fernández ME, Abadie B, Damschroder LJ (2019). Choosing implementation strategies to address contextual barriers: diversity in recommendations and future directions. Implement Sci.

[23] Bunger AC, Powell BJ, Robertson HA, MacDowell H, Birken SA, Shea C (2017). Tracking implementation strategies: a description of a practical approach and early findings. Health Res Policy Syst.

[24] Proctor EK, Powell BJ, McMillen JC (2013). Implementation strategies: recommendations for specifying and reporting. Implement Sci.

[25] Prochaska JO, CC DC (1983). Stages and processes of self-change of smoking: toward an integrative model of change. J Consult Clin Psychol.

[26] Migneault J, Adams T, Read J (2005). Application of the Transtheoretical Model to substance abuse: historical development and future directions. Drug Alcohol Rev.

[27] Nidecker M, DiClemente CC, Bennett ME, Bellack AS (2008). Application of the Transtheoretical Model of change: psychometric properties of leading measures in patients with co-occurring drug abuse and severe mental illness. Addict Behav.

[28] Prochaska JM, Cohen FC, Gomes SO, Laforge RG, Eastwood AL (2004). The transtheoretical model of change for multi-level interventions for alcohol abuse on campus. J Alcohol Drug Educ.

[29] Velicer WF, Norman GJ, Fava JL, Prochaska JO (1999). Testing 40 predictions from the transtheoretical model. Addict Behav.

[30] Aveyard P, Massey L, Parsons A, Manaseki S, Griffin C (2009). The effect of Transtheoretical Model based interventions on smoking cessation. Soc Sci Med.

[31] Kushnir V, Godinho A, Hodgins DC, Hendershot CS, Cunningham JA (2016). Motivation to quit or reduce gambling: associations between self-determination theory and the Transtheoretical Model of change. J Addict Dis.

[32] Prochaska JM, Prochaska JO, Levesque DA (2001). A transtheoretical approach to changing organizations. Admin Pol Ment Health.

[33] Berry TR, Plotnikoff RC, Raine K, Anderson D, Naylor PJ (2007). An examination of the stages of change construct for health promotion within organizations. J Health Organ Manag.

[34] Suryadevara KM. Assessing climate for systems improvement initiatives in healthcare [dissertation]. Kingston, Rhode Island: University of Rhode Island; 2015. Available from: http://ovidsp.ovid.com/ovidweb.cgi?T=JS&PAGE=reference&D=psyc13&NEWS=N&AN=2016-26527-296.

[35] Theberge-Smith P. Organizational readiness to change for professional collaboration (thesis). Nashua, New Hampshire: Rivier University; 2018. Available from: https://www.proquest.com/openview/5e7cb29866c212ae426a9e1443f77a8e/1?pq-origsite=gscholar&cbl=18750&diss=y.

[36] Lyons JB, Swindler SD, Offner A (2009). The impact of leadership on change readiness in the US military. J Chang Manag.

[37] Levesque DA, Prochaska JM, Prochaska JO (1999). Stages of change and integrated service delivery. Consult Psychol J Pract Res.

[38] Smathers C, Washburn L, Toomey M, Johannes E, Johnston K (2018). Organizational readiness to engage in policy, system, and environment changes supporting positive youth development for health: case studies from the cooperative extension system framed by the Transtheoretical Model. J Hum Sci Ext.

[39] Grimolizzi-Jansen CJ (2018). Organizational change: effect of motivational interviewing on readiness to change. J Chang Manag.

[40] Prochaska JM (2007). The transtheoretical model applied to the community and the workplace. J Health Psychol.

[41] Sackman H (1974). Delphi assessment: expert opinion, forecasting and group process. Vol. R-1283-PR, United States Air Force Project RAND.

[42] Trevelyan EG, Robinson N (2015). Delphi methodology in health research: how to do it?. Eur J Integr Med.

[43] Powell C (2003). The Delphi technique: Myths and realities. J Adv Nurs.

[44] Custer RL, Scarcella JA, Stewart BR (1999). The modified Delphi technique - a rotational modification. J Vocat Tech Educ.

[45] Avella JR (2016). Delphi panels: Research design, procedures, advantages, and challenges. Int J Dr Stud.

[46] Oostendorp LJ, Durand M-A, Lloyd A, Elwyn G (2015). Measuring organisational readiness for patient engagement (MORE): an international online Delphi consensus study. BMC Health Serv Res.

[47] Attieh R, Gagnon M-P, Estabrooks CA, Légaré F, Ouimet M, Vazquez P (2014). Organizational readiness for knowledge translation in chronic care: a Delphi study. BMC Health Serv Res.

[48] Domlyn AM, Wandersman A (2019). Community coalition readiness for implementing something new: using a Delphi methodology. J Community Psychol.

[49] Minas H, Jorm AF (2010). Where there is no evidence: use of expert consensus methods to fill the evidence gap in low-income countries and cultural minorities. Int J Ment Health Syst.

[50] Donohoe H, Stellefson M, Tennant B (2012). Advantages and limitations of the e-Delphi technique: implications for health education researchers. Am J Health Educ.

[51] Diamond IR, Grant RC, Feldman BM, Pencharz PB, Ling SC, Moore AM, et al. Defining consensus: a systematic review recommends methodologic criteria for reporting of Delphi studies. J Clin Epidemiol. 2014;67(4):401–9. 10.1016/j.jclinepi.2013.12.002. 10.1016/j.jclinepi.2013.12.002 24581294

[52] Levesque DA, Prochaska JM, Prochaska JO, Dewart S, Hamby LS, Weeks WB (2001). Organizational stages and processes of change for continuous quality improvement in health care. Consult Psychol J Pract Res.

[53] Perry CK, Damschroder LJ, Hemler JR, Woodson TT, Ono SS, Cohen DJ (2019). Specifying and comparing implementation strategies across seven large implementation interventions: a practical application of theory. Implement Sci.

[54] Rogers EM (2003). Diffusion of innovation.

[55] Laforge RG, Velicer WF, Richmond RL, Owen N (1999). Stage distributions for five health behaviors in the United States and Australia. Prev Med (Baltim).

[56] Keith RE, Crosson JC, O’malley AS, Cromp D, Taylor EF. Using the Consolidated Framework for Implementation Research (CFIR) to produce actionable findings: a rapid-cycle evaluation approach to improving implementation. Implement Sci. 2017;12(15) [cited 2018 Feb 11]. Available from: https://implementationscience.biomedcentral.com/track/pdf/10.1186/s13012-017-0550-7?site=implementationscience.biomedcentral.com. 10.1186/s13012-017-0550-7 PMC5303301 28187747

[57] Vakola M (2014). What’s in there for me? Individual readiness to change and the perceived impact of organizational change. Leadersh Org Dev J.

[58] Aarons GA, Cafri G, Lugo L (2012). Expanding the domains of attitudes towards evidence-based practice: the evidence based practice attitude scale-50. Admin Pol Ment Health.

[59] Dave B, Bradley H, M. SG, G. L de PS (2019). Facilitating change through groups: formation of collective attitudes toward change. Research in organizational change and development.

[60] Lehman WEK, Greener JM, Simpson DD (2002). Assessing organizational readiness for change. J Subst Abus Treat.

[61] Campbell SM, Shield T, Rogers A (2004). How do stakeholder groups vary in a Delphi technique about primary mental health care and what factors influence their ratings?. Qual Saf Health Care.

