# Supplementary material for: Enhancing organizational readiness for implementation: constructing a typology of readiness-development strategies using a modified Delphi process
Source: Implement Sci. 2021 Jun 10;16:61. doi: 10.1186/s13012-021-01132-0 (PMC8194182; doi:10.1186/s13012-021-01132-0)
Supplement: Supplementary file 1 — Additional file 1. ORI Delphi_Supplement 1_CREDES Reporting Checklist.PDF – CREDES reporting checklist for a Delphi study. [file 13012_2021_1132_MOESM1_ESM.pdf]

Additional file 1: CREDES CHECKLIST FOR REPORTING DELPHI STUDIES

Jünger, S., Payne, S. A., Brine, J., Radbruch, L., & Brearley, S. G. (2017). Guidance on Conducting and REporting DElphi Studies (CREDES) in palliative care: Recommendations based on a methodological systematic review. *Palliative Medicine*, *31*(8), 684–706. https://doi.org/10.1177/0269216317690685

No.	Topic	Item	Reported in pages	Comments
**Rationale for the choice of the Delphi technique**				
1.	Justification	The choice of the Delphi technique as a method of systematically collating expert consultation and building consensus needs to be well justified. When selecting the method to answer a particular research question, it is important to keep in mind its constructivist nature	4-5, 7	Knowledge gap and choice of methodology as a solution explained in the Background section. The Modified Delphi Method and rationale explained in the Methods section.
**Planning and Design**				
2.	Planning and process	The Delphi technique is a flexible method and can be adjusted to the respective research aims and purposes. Any modifications should be justified by a rationale and be applied systematically and rigorously	7-11	The Delphi modifications and specific structure of each round are explained in the Methods section. Figure 1 presents a flow chart describing the process
3.	Definition of consensus	Unless not reasonable due to the explorative nature of the study, an a priori criterion for consensus should be defined. This includes a clear and transparent guide for action on (a) how to proceed with certain items or topics in the next survey round, (b) the required threshold to terminate the Delphi process and (c) procedures to be followed when consensus is (not) reached after one or more iterations	7-11	A-priori consensus criterion and a clear guide for building the consensus through each round are described.
**Study Conduct**				
4.	Informational input	All material provided to the expert panel at the outset of the project and throughout the Delphi process should be carefully reviewed and piloted in advance in order to examine the effect on experts' judgements and to prevent bias	22	The questionnaires were piloted by two implementation experts mentioned in the Acknowledgement section.

5.	Prevention of bias.	Researchers need to take measures to avoid directly or indirectly influencing the experts' judgements. If one or more members of the research team have a conflict of interest, entrusting an independent researcher with the main coordination of the Delphi study is advisable	8, 21	IRB provision as well as measures for ensuring independent rating by all participants are described. The authors declare they had no competing interests
6.	Interpretation and processing of results.	Consensus does not necessarily imply the 'correct' answer or judgement; (non)consensus and stable disagreement provide informative insights and highlight differences in perspectives concerning the topic in question	7-11	Changes in agreement between the rounds are described. Figure 1 presents the steps for processing the results
7.	External validation.	It is recommended to have the final draft of the resulting guidance on best practice in palliative care reviewed and approved by an external board or authority before publication and dissemination	22	Authors 2-5 supervised the study and provided feedback on the manuscript as part of their role as the first author's dissertation committee members.
**Reporting**				
8.	Purpose and rationale.	The purpose of the study should be clearly defined and demonstrate the appropriateness of the use of the Delphi technique as a method to achieve the research aim. A rationale for the choice of the Delphi technique as the most suitable method needs to be provided	7, 14-15	Choice of method in relation to the study aims is described in the Methods section as well as in the opening of the Discussion.
9.	Expert panel.	Criteria for the selection of experts and transparent information on recruitment of the expert panel, sociodemographic details including information on expertise regarding the topic in question, (non)response and response rates over the ongoing iterations should be reported	6-7, 20	Delphi Participants section + Contributors section
10.	Description of the methods	The methods employed need to be comprehensible; this includes information on preparatory steps (How was available evidence on the topic in question synthesized?), piloting of material and survey instruments, design of the survey instrument(s), the number and design of survey rounds, methods of data analysis, processing and	6-11	Methods section

		synthesis of experts' responses to inform the subsequent survey round and methodological decisions taken by the research team throughout the process		
11.	Procedure.	Flow chart to illustrate the stages of the Delphi process, including a preparatory phase, the actual 'Delphi rounds', interim steps of data processing and analysis, and concluding steps	11	Figure 1
12.	Definition and attainment of consensus	It needs to be comprehensible to the reader how consensus was achieved throughout the process, including strategies to deal with non-consensus	8-11	All procedures are described per round and for the final analysis.
13.	Results.	Reporting of results for each round separately is highly advisable in order to make the evolving of consensus over the rounds transparent. This includes figures showing the average group response, changes between rounds, as well as any modifications of the survey instrument such as deletion, addition or modification of survey items based on previous rounds	11-14	Results section including Table 1 that presents the final typology and reference to Supplement 2 that presents the final agreement level per strategy
14.	Discussion of limitations	Reporting should include a critical reflection of potential limitations and their impact of the resulting guidance	18-19	
15.	Adequacy of conclusions	The conclusions should adequately reflect the outcomes of the Delphi study with a view to the scope and applicability of the resulting practice guidance	14-19	Discussion section
16.	Publication and dissemination	The resulting guidance on good practice in palliative care should be clearly identifiable from the publication, including recommendations for transfer into practice and implementation. If the publication does not allow for a detailed presentation of either the resulting practice guidance or the methodological features of the applied Delphi technique, or both, reference to a more detailed presentation elsewhere should be made (e.g. availability of the full guideline from the authors or online; publication of a separate paper reporting on methodological details and	14-20	This is only partially relevant for the results of this study, as we offer an initial framework that should be further tested and elaborated. Recommendations for future studies are included in the Discussion and Conclusion.

		particularities of the process (e.g. persistent disagreement and controversy on certain issues). A dissemination plan should include endorsement of the guidance by professional associations and health care authorities to facilitate implementation		
