# Supplementary material for: Enhancing organizational readiness for implementation: constructing a typology of readiness-development strategies using a modified Delphi process
Source: Implement Sci. 2021 Jun 10;16:61. doi: 10.1186/s13012-021-01132-0 (PMC8194182; doi:10.1186/s13012-021-01132-0)
Supplement: Supplementary file 2 — Additional file 2. ORI Delphi_Supplement 2_Results.PDF – A summary of the results from Round 2 and Round 3. [file 13012_2021_1132_MOESM2_ESM.pdf]

Supplement 2 Strategies assignment and levels of agreement per category (N=73)

Strategy	Round 2	Round 3	Description	ORI-1	ORI-2	ORI-3	Not ORI related
Access new funding [b]	✓		Access new or existing money to facilitate the implementation	Low	High	High	
Alter incentive / allowance structures [b]	✓		Work to incentivize the adoption and implementation of the clinical innovation	Low	High	High	
Alter patient / consumer fees [b]	✓		Create fee structures where patients/consumers pay less for preferred treatments (the clinical innovation) and more for less-preferred treatments	Low	Low	High	Low
Assess for readiness and identify barriers and facilitators [c]	✓	✓	Assess various aspects of an organization to determine its degree of readiness to implement, barriers that may impede implementation, and strengths that can be used in the implementation effort	Medium	High	Medium	
Audit and provide feedback [a]			Collect and summarize clinical performance data over a specified time period and give it to clinicians and administrators to monitor, evaluate, and modify provider behavior				Medium
Build a coalition [b]	✓		Recruit and cultivate relationships with partners in the implementation effort	Low	High	High	
Capture and share local knowledge [a]			Capture local knowledge from implementation sites on how implementers and clinicians made something work in their setting and then share it with other sites				Medium
Centralize technical assistance [b]	✓		Develop and use a centralized system to deliver technical assistance focused on implementation issues		Low	High	Low
Change accreditation or membership requirements [b]	✓		Strive to alter accreditation standards so that they require or encourage use of the clinical innovation. Work to alter membership organization requirements so that those who want to affiliate with the organization are encouraged or required to use the clinical innovation	Low	Low	High	Low
Change liability laws [d]	✓	✓	Participate in liability reform efforts that make clinicians more willing to deliver the clinical innovation	Medium	Medium	Medium	
Change physical structure and equipment [b]	✓		Evaluate current configurations and adapt, as needed, the physical structure and/or equipment (e.g., changing the layout of a room, adding equipment) to best	Low	Low	High	Low

Strategy	Round 2	Round 3	Description	ORI-1	ORI-2	ORI-3	Not ORI related
			accommodate the targeted innovation				
Change record system [b]	✓		Change records systems to allow better assessment of implementation or clinical outcomes	Low	Low	High	Low
Change service sites [a]			Change the location of clinical service sites to increase access Conduct				Medium
Conduct cyclical small tests of change [a]			Implement changes in a cyclical fashion using small tests of change before taking changes system-wide. Tests of change benefit from systematic measurement, and results of the tests of change are studied for insights on how to do better. This process continues serially over time, and refinement is added with each cycle				High
Conduct educational meetings [b]	✓		Hold meetings targeted toward different stakeholder groups (e.g., providers, administrators, other organizational stakeholders, and community, patient/consumer, and family stakeholders) to teach them about the clinical innovation	High	High	High	
Conduct educational outreach visits [c]	✓	✓	Have a trained person meet with providers in their practice settings to educate providers about the clinical innovation with the intent of changing the provider's practice	Medium	High	Medium	
Conduct local consensus discussions [b]	✓		Include local providers and other stakeholders in discussions that address whether the chosen problem is important and whether the clinical innovation to address it is appropriate	High	High	Low	
Conduct local needs assessment [b]	✓		Collect and analyze data related to the need for the innovation	High	High	Low	
Conduct ongoing training [a]			Plan for and conduct training in the clinical innovation in an ongoing way				Medium
Create a learning collaborative [a]			Facilitate the formation of groups of providers or provider organizations and foster a collaborative learning environment to improve implementation of the clinical innovation				Medium
Create new clinical teams [b]	✓		Change who serves on the clinical team, adding different disciplines and different skills to make it more likely that the clinical innovation is delivered (or is more successfully delivered)	Low	Low	High	Low

Strategy	Round 2	Round 3	Description	ORI-1	ORI-2	ORI-3	Not ORI related
Create or change credentialing and/or licensure standards [b]	✓		Create an organization that certifies clinicians in the innovation or encourage an existing organization to do so. Change governmental professional certification or licensure requirements to include delivering the innovation. Work to alter continuing education requirements to shape professional practice toward the innovation	Low	Low	High	Low
Develop a formal implementation blueprint [b]	✓		Develop a formal implementation blueprint that includes all goals and strategies. The blueprint should include the following: High) aim/purpose of the implementation; Medium) scope of the change (e.g., what organizational units are affected); Low) timeframe and milestones; and 4) appropriate performance/progress measures. Use and update this plan to guide the implementation effort over time	Low	Low	High	
Develop academic partnerships [d]	✓	✓	Partner with a university or academic unit for the purposes of shared training and bringing research skills to an implementation project	Medium	Medium	Medium	
Develop an implementation glossary [c]	✓	✓	Develop and distribute a list of terms describing the innovation, implementation, and stakeholders in the organizational change	Medium	Low	High	Low
Develop and implement tools for quality monitoring [b]	✓		Develop, test, and introduce into quality-monitoring systems the right input—the appropriate language, protocols, algorithms, standards, and measures (of processes, patient/consumer outcomes, and implementation outcomes) that are often specific to the innovation being implemented	Low	Low	High	
Develop and organize quality monitoring systems [c]	✓	✓	Develop and organize systems and procedures that monitor clinical processes and/or outcomes for the purpose of quality assurance and improvement	Low	Medium	High	
Develop disincentives [c]	✓	✓	Provide financial disincentives for failure to implement or use the clinical innovations	Low	Medium	High	
Develop educational materials [c]	✓	✓	Develop and format manuals, toolkits, and other supporting materials in ways that make it easier for stakeholders to learn about the innovation and for	High	Medium	Medium	

Strategy	Round 2	Round 3	Description	ORI-1	ORI-2	ORI-3	Not ORI related
			clinicians to learn how to deliver the clinical innovation				
Develop resource sharing agreements [b]	✓		Develop partnerships with organizations that have resources needed to implement the innovation		Low	High	
Distribute educational materials [c]	✓	✓	Distribute educational materials (including guidelines, manuals, and toolkits) in person, by mail, and/or electronically	Medium	Medium	High	
Facilitate relay of clinical data to providers [a]			Provide as close to real-time data as possible about key measures of process/outcomes using integrated modes/channels of communication in a way that promotes use of the targeted innovation				High
Facilitation [a]			A process of interactive problem solving and support that occurs in a context of a recognized need for improvement and a supportive interpersonal relationship				Medium
Fund and contract for clinical innovation [c]	✓	✓	Governments and other payers of services issue requests for proposals to deliver the innovation, use contracting processes to motivate providers to deliver the clinical innovation, and develop new funding formulas that make it more likely that providers will deliver the innovation	Medium	High	Medium	Low
Identify and prepare champions [c]	✓	✓	Identify and prepare individuals who dedicate themselves to supporting, marketing, and driving through an implementation, overcoming indifference or resistance that the intervention may provoke in an organization	Medium	High	Medium	
Identify early adopters [c]	✓	✓	Identify early adopters at the local site to learn from their experiences with the practice innovation	Medium	High	Medium	
Increase demand [d]	✓	✓	Attempt to influence the market for the clinical innovation to increase competition intensity and to increase the maturity of the market for the clinical innovation	Medium	Medium	Medium	
Inform local opinion leaders [c]	✓	✓	Inform providers identified by colleagues as opinion leaders or "educationally influential" about the clinical innovation in the hopes that they will influence colleagues to adopt it	High	High	Low	
Intervene with patients/consumers to enhance uptake & adherence [a]			Develop strategies with patients to encourage and problem solve around adherence				Medium

Strategy	Round 2	Round 3	Description	ORI-1	ORI-2	ORI-3	Not ORI related
Involve executive boards [d]	✓	✓	Involve existing governing structures (e.g., boards of directors, medical staff boards of governance) in the implementation effort, including the review of data on implementation processes	Medium	Medium	Medium	
Involve patients/consumers and family members [c]	✓	✓	Engage or include patients/consumers and families in the implementation effort	Medium	Medium	High	
Make billing easier [c]	✓	✓	Make it easier to bill for the clinical innovation	Low	Medium	High	
Make training dynamic [b]	✓		Vary the information delivery methods to cater to different learning styles and work contexts, and shape the training in the innovation to be interactive	Low	Low	High	
Mandate change [d]	✓	✓	Have leadership declare the priority of the innovation and their determination to have it implemented	Medium	Medium	Medium	
Model and simulate change [c]	✓	✓	Model or simulate the change that will be implemented prior to implementation	Low	Medium	High	
Obtain and use patients/consumers and family feedback [a]			Develop strategies to increase patient/consumer and family feedback on the implementation effort				Medium
Obtain formal commitments [c]	✓	✓	Obtain written commitments from key partners that state what they will do to implement the innovation		Medium	High	
Organize clinician implementation team meetings [a]			Develop and support teams of clinicians who are implementing the innovation and give them protected time to reflect on the implementation effort, share lessons learned, and support one another's learning				Medium
Place innovation on fee for service lists / formularies [c]	✓	✓	Work to place the clinical innovation on lists of actions for which providers can be reimbursed (e.g., a drug is placed on a formulary, a procedure is now reimbursable)	Medium	Medium	High	
Prepare patients / consumers to be active participants [c]	✓	✓	Prepare patients/consumers to be active in their care, to ask questions, and specifically to inquire about care guidelines, the evidence behind clinical decisions, or about available evidence-supported treatments	Medium	Medium	High	
Promote adaptability [b]	✓		Identify the ways a clinical innovation can be tailored to meet local needs and clarify which elements of the innovation must be maintained to preserve fidelity	Low	High	High	

Strategy	Round 2	Round 3	Description	ORI-1	ORI-2	ORI-3	Not ORI related
Promote network weaving [c]	✓	✓	Identify and build on existing high-quality working relationships and networks within and outside the organization, organizational units, teams, etc. to promote information sharing, collaborative problem-solving, and a shared vision/goal related to implementing the innovation	Low	Medium	High	
Provide clinical supervision [a]			Provide clinicians with ongoing supervision focusing on the innovation. Provide training for clinical supervisors who will supervise clinicians who provide the innovation				High
Provide local technical assistance [a]			Develop and use a system to deliver technical assistance focused on implementation issues using local personnel				Medium
Provide ongoing consultation [a]			Provide ongoing consultation with one or more experts in the strategies used to support implementing the innovation				High
Purposely reexamine the implementation [a]			Monitor progress and adjust clinical practices and implementation strategies to continuously improve the quality of care				High
Recruit, designate and train for leadership [b]	✓		Recruit, designate, and train leaders for the change effort	Low	High	High	
Remind clinicians [a]			Develop reminder systems designed to help clinicians to recall information and/or prompt them to use the clinical innovation				High
Revise professional roles [b]	✓		Shift and revise roles among professionals who provide care, and redesign job characteristics	Low	Low	High	Low
Shadow other experts [b]	✓		Provide ways for key individuals to directly observe experienced people engage with or use the targeted practice change/innovation	Low	High	High	
Stage implementation scale up [b]	✓		Phase implementation efforts by starting with small pilots or demonstration projects and gradually move to a system wide rollout	Low	Low	High	Low
Start a dissemination organization [d]	✓	✓	Identify or start a separate organization that is responsible for disseminating the clinical innovation. It could be a for-profit or non-profit organization	Low	Low	Medium	Medium
Tailor strategies [c]	✓	✓	Tailor the implementation strategies to address barriers and leverage facilitators that were	Low	Medium	High	

Strategy	Round 2	Round 3	Description	ORI-1	ORI-2	ORI-3	Not ORI related
			identified through earlier data collection				
Use advisory boards and workgroups [b]	✓		Create and engage a formal group of multiple kinds of stakeholders to provide input and advice on implementation efforts and to elicit recommendations for improvements	Low	High	High	
Use an implementation adviser [c]	✓	✓	Seek guidance from experts in implementation	Medium	High	High	
Use capitated payments [a]			Pay providers or care systems a set amount per patient/consumer for delivering clinical care				Medium
Use data experts [c]	✓	✓	Involve, hire, and/or consult experts to inform management on the use of data generated by implementation efforts	Low	Medium	High	Low
Use data warehousing techniques [c]	✓	✓	Integrate clinical records across facilities and organizations to facilitate implementation across systems	Low	Medium	High	Low
Use mass media [d]	✓	✓	Use media to reach large numbers of people to spread the word about the clinical innovation	Medium	Medium	Medium	
Use other payment schemes [a]			Introduce payment approaches (in a catch-all category)				Medium
Use train the trainer strategies [c]	✓	✓	Train designated clinicians or organizations to train others in the clinical innovation	Low	Medium	High	Low
Visit other sites [c]	✓	✓	Visit sites where a similar implementation effort has been considered successful	Low	High	Medium	
Work with educational institutions [c]	✓	✓	Encourage educational institutions to train clinicians in the innovation	Medium	Medium	High	Low
**TOTAL**	**55**	**32**					

High-level agreement (≥60%), Medium-level agreement (30-59%), Low-level agreement (<30%)
[a] Strategies excluded after Round 1 (*n*=18)
[b] Strategies included in the typology after Round 2 (*n*=23)
[c] Strategies included in the typology after Round 3 (*n*=25)
[d] Strategies excluded after Round 3 (*n*=7)
